# Thiopurines in Inflammatory Bowel Disease. How to Optimize Thiopurines in the Biologic Era?

**DOI:** 10.3389/fmed.2021.681907

**Published:** 2021-07-16

**Authors:** Carla J. Gargallo-Puyuelo, Viviana Laredo, Fernando Gomollón

**Affiliations:** ^1^Department of Gastroenterology, University Clinic Hospital Lozano Blesa, Zaragoza, Spain; ^2^Department of Medicine, Psychiatry and Dermatology, University of Zaragoza, Zaragoza, Spain; ^3^Institute for Health Research Aragón (IIS Aragón), Zaragoza, Spain; ^4^Centro de Investigación Biomédica en Red, Enfermedades Hepáticas y Digestivas, Madrid, Spain

**Keywords:** thiopurines, inflammatory bowel disease, pharmacogenomics, toxicity, indications, optimize

## Abstract

Thiopurines have been a cornerstone in the treatment of inflammatory bowel disease (IBD). Although they have been used for more than 50 years, there are still some unsolved issues about their efficacy and, also, some safety concerns, mainly the risk of myelosuppression and life-threatening lymphoproliferative disorders. Furthermore, the development of biological therapy raises the question whether there is still a role for thiopurines in the IBD treatment algorithm. On the other hand, limited cost and wide availability make thiopurines a reasonable option in settings of limited resources and increasing prevalence of IBD. In fact, there is a growing interest in optimizing thiopurine therapy, since pharmacogenomic findings suggest that a personalized approach based on the genotyping of some molecules involved in its metabolism could be useful to prevent side effects. Polymorphisms of thiopurine methyltransferase enzyme (TPMT) that result in low enzymatic activity have been associated with an increased risk of myelotoxicity, especially in Caucasians; however, in Asians it is assumed that the variants of nudix hydrolase 15 (NUDT15) are more relevant in the development of toxicity. Age is also important, since in elderly patients the risk of complications seems to be increased. Moreover, the primo-infection of Epstein Barr virus and cytomegalovirus under thiopurine treatment has been associated with severe lymphoproliferative disorders. In addition to assessing individual characteristics that may influence thiopurines treatment outcomes, this review also discusses other strategies to optimize the therapy. Low-dose thiopurines combined with allopurinol can be used in hypermethylators and in thiopurine-related hepatotoxicity. The measurement of metabolites could be useful to assess compliance, identify patients at risk of adverse events and also facilitating the management of refractory patients. Thioguanine is also a rescue therapy in patients with toxicity related to conventional thiopurine therapy. Finally, the current indications for thiopurines in monotherapy or in combination with biologics, as well as the optimal duration of treatment, are also reviewed.

## Introduction

Inflammatory bowel disease (IBD) includes mainly two chronic disorders affecting the gastrointestinal tract, Crohn's disease (CD) and ulcerative colitis (UC), and it has a worldwide distribution (1). The medical treatment is based on 5-aminosalicylates, corticosteroids, immunomodulators (thiopurines and methotrexate) and biologics (2, 3).

Thiopurines (azathioprine, mercaptopurine and thioguanine) are antimetabolites of purines which have been a cornerstone in the treatment of IBD for more than 50 years (4). In spite of that, there is still lack of evidence about its efficacy in some scenarios. Firstly, guidelines do not recommend using thiopurines (TP) as induction therapy (2, 3, 5–7). In CD most evidence comes from studies comparing azathioprine (AZA) and placebo (8); and there is only one randomized controlled trial comparing AZA and biologic therapy (infliximab), concluding that AZA was inferior (9). Although the quality of the studies has been questioned, the evidence for AZA as induction therapy in UC is also absent (7, 10). In maintaining of remission, there are Cochrane reviews of randomized controlled trials for both, CD and UC, demonstrating a superiority of AZA against placebo; however, the quality of evidence is again low, especially in UC (11, 12). Another common indication is the prevention of post-surgical relapse, but despite the fact that it seems to be superior to placebo, there is a wide heterogeneity in the designs of studies and in one small randomized trial comparing AZA with biologics (adalimumab) there were no differences in efficacy between both treatments while in other study adalimumab was superior (5, 13, 14). Finally, the evidence supporting combination therapy of AZA with biologics relays mainly on two prospective trials in which combination therapy was superior to monotherapy in CD and UC (9, 15). Despite the superiority, the appropriate duration of combination therapy is still unknown (5, 7).

In addition to unsolved efficacy issues, safety concerns may also limit the use of TP in clinical practice. The rate of adverse events is up to 25% in some studies and nearly 20% of patients have to discontinue the treatment (16). Some strategies, as periodic blood tests, determination of genetic polymorphisms and metabolites measurement, are useful to decrease the risk of some side effects such as myelotoxicity, but it cannot be prevented in all cases and it can occur at any time of the treatment (17). Although it could be a serious adverse event, the risk of death due to myelotoxicity is relatively low (1%) (18). Other uncommon life-threatening hematological conditions, such as hemophagocytic lymphohistiocytosis (HLH) and other lymphoproliferative disorders have also been associated with TP (19). Pancreatitis and hepatotoxicity are other limiting side effects related to TP (20, 21).

Despite efficacy issues, toxicity risks and the availability of other therapeutic options, there are some arguments in favor of optimizing TP. Firstly, the epidemiological evolution of IBD is changing and pediatric onset of the disease is becoming more common in some Western countries (1, 22), which implies a longer evolution of the disease in these patients and, probably, the need for different IBD treatments during their life; especially considering that the course of the disease seems to be more aggressive (23). In spite of the availability of many therapeutic options for IBD, there are still some refractory patients who will eventually need surgery (24); therefore, optimizing medical treatment before escalation seems a reasonable option. Furthermore, due to the increasing prevalence of IBD in Western countries, the number of patients in IBD units and, consequently, the treatment-related costs are expected to increase (1, 25). TP are cheaper compared to biologics; in fact, in some countries, before the biologics, the cost of drugs represented 25% of the IBD care cost and, after them, the cost has increased from 30 to 70% (26). Finally, the overall efficacy of TP has been demonstrated for many years and, in general, patients who respond to these drugs tend to maintain a long remission (4, 27). Clinical experience with TP also helps to manage most side effects, and those that are life-threatening are uncommon.

## How to Improve Efficiency of Thiopurines?

### Using the Treatment in Selected Patients Depending on Individual Characteristics

#### Pharmacogenomics

**Figure 1:**
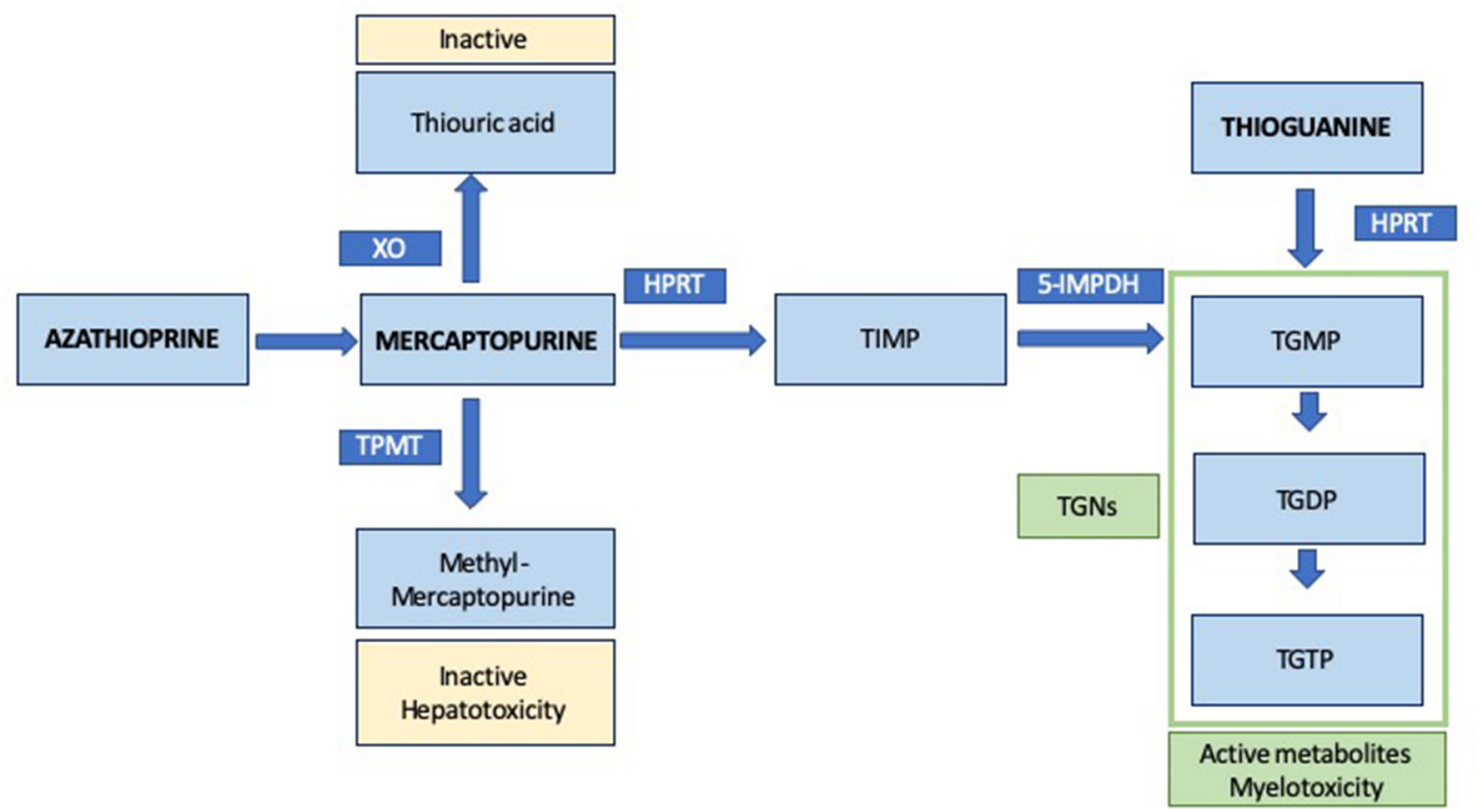
Simplified metabolism of thiopurines. XO, xanthine oxidase; TPMT, thiopurine methyltransferase; HPRT, hypoxanthine-guanine phosphoribosyltransferase; 5-IMPDH, 5- inosine monophosphate dehydrogenase; TIMP, thiosine monophosphate; TGNs, thioguanine nucleotides; TGMP, thioguanine monophosphate; TGDP, thioguanine diphosphate; TGTP, thioguanine triphosphate.

AZA is a prodrug and, after a non-enzymatic change, 88% of it is converted into mercaptopurine (MP), that can be metabolized through different pathways into another active and inactive metabolites, as shown in Figure 1 (27, 28). The thiopurine methyltransferase enzyme (TPMT) methylates MP into methylmercaptopurine (MMP), an inactive metabolite associated with some adverse events, mainly hepatotoxicity. MP can also be oxidized by xanthine oxidase into thiouric acid (TUA), another inactive degradation product. However, MP can be converted by hypoxanthine-guanine phosphoribosyltransferase (HPRT) into thiosine monophosphate (TIMP), which can also be transformed by 5- inosine monophosphate dehydrogenase (5-IMPDH) into thioguanine monophosphate (TGMP) and, then, into thioguanine diphosphate (TGDP) and triphosphate (TGTP) (28). Thioguanine (TG) is also metabolized by HPRT into TGMP. TGMP, TGDP and TGTP are thioguanine nucleotides (TGNs) and the active metabolites of AZA, responsible for the efficacy and myelotoxicity of TP. These nucleotides antagonize the endogenous purines and incorporate into cellular RNA-DNA, inhibiting cellular proliferation. Other mechanism of action includes inhibition of Rac1 activation with costimulation of CD28 leading to T cell apoptosis (29). The main action of TPMT enzyme is to methylate MP, TG, TIMP and TGMP; so they become inactive products, TGNs synthesis decreases and, subsequently, TP are less effective (30). Efficacy and side effects are consequence of a tight balance between the pathways that activate and inactive TP and a wide inter-individual variability has been described in this setting (31).

Currently, more and more studies highlight the role of pharmacogenomics in optimizing TP (31–33). Polymorphisms of TPMT, nudix hydrolase 15 (NUDT15), alpha-ketoglutarate dependent dioxygenase (FOT), class II HLA and inosine triphosphate pyrophosphatase (ITPA) have been associated with an increased risk of adverse events (Table 1).

**Table 1:** Most important genetic variants associated with thiopurine toxicity.

**Genetic variant**	**Functional consequence**	**Clinical consequence**
TPMT*2 (rs1800462) TPMT*3C (rs1142345) TPMT*3A: contains *3B (rs1800460) and *3C (rs1142345)	Low TMPT enzymatic activity	Risk of myelotoxicity
NUDT15 p.Arg139Cys or c415C>T (rs116855232)	Low NUDT15 enzymatic activity	Risk of myelotoxicity
NUDT15 p.Val18_Val19insGlyVal allele	Low NUDT15 enzymatic activity	Risk of myelotoxicity
Class II HLA polymorphism (rs2647087)	Unclear	Risk of pancreatitis
ITPA 94C > A (rs1127354)	Low enzymatic activity	Inconclusive data about increased risk of side effects
FTO Ala134Thr (rs79206939)	Low enzymatic activity	Leukopenia

##### Thiopurine Methyltransferase

Patients with increased TPMT activity are called “hypermethylators” or “non-responders” because they mainly produce methylated inactivated products of AZA with very low amounts of active metabolites (34). On the other hand, patients with low-activity in both alleles of TPMT gene mainly produce active metabolites by increasing IMPDH pathway and are at risk of severe adverse events, especially myelotoxicity. The TPMT alleles can be classified into functional (^*^1) or non-functional (^*^2, ^*^3A, ^*^3B, ^*^3C, ^*^4) (35). There are many different polymorphisms associated with low TPMT activity but the most important are TPMT^*^2, TPMT^*^3A and TPMT^*^3C, as they represent 60–95% deficient alleles in most populations (31). Depending on genotype, patients could be classified into homozygous of high activity alleles (two or more functional alleles), heterozygous with intermediate activity (one functional allele and one non-functional) and homozygous with low activity (two non-functional alleles). In Caucasians, 0.3% are homozygous for low activity alleles, 11% are heterozygous and 89% are homozygous for high activity alleles (36). Based on the genotype, a full dose of TP is recommended in high activity homozygous, a 50% dose-reduction in heterozygotes, and avoiding treatment in low activity homozygous (35, 37). TPMT activity can be measured using phenotype in red blood cells or genotype, based on the analysis of polymorphisms associated with low TPMT activity (38). Genotype appears to be superior to phenotype, as it better identifies heterozygous with similar rates by misclassifying TPMT defectives (39). On the other hand, one advantage of TPMT phenotype, rather than genotype, is that within one genotype there can be significant variation in phenotypes, allowing for further individualization of dosing.

Despite the theoretical utility of TPMT genotyping, the TOPIC trial did not find an advantage of this strategy to reduce the incidence of myelotoxicity (37), except in heterozygotes with high-risk variants of TPMT, while the TARGET trial did not find a benefit in this subgroup of patients (40). The correct management of heterozygous is also controversial. Studies suggest that 30–60% of patients with intermediate TPMT activity will develop myelotoxicity under full dose of thiopurines, therefore the guidelines recommend a dose-reduction in heterozygotes; however, if we guide treatment only by TPMT activity, 40% of patients will be undertreated (31, 32). Furthermore, only 25% of myelosuppression can be explained by a TPMT deficiency (41).

The benefit of routinely testing TPMT is unclear (42). Some societies, such as the *American Gastroenterological Association* (43) and the *British Society of Gastroenterology* (6), recommend testing TPMT routinely before starting TP, but with low quality of evidence, while others, such as the *Spanish Working Group on Crohn's and Ulcerative Colitis* (GETECCU) or the *European Crohn's and Colitis Organization* (ECCO) support this recommendation, but suggest that is not essential before starting treatment (3, 5). Regarding the cost of routine TPMT testing, some authors suggest that it is cost-effective (33, 38) and others conclude that genotyping is a cost-neutral strategy (44). If available, it seems reasonable to test TPMT before starting TP; however, it may also depend on the prevalence of homozygous and heterozygous in each population (18). Moreover, periodic blood tests are mandatory throughout treatment because the risk of myelotoxicity does not disappear (42).

##### Nudix Hydrolase 15

The incidence of leukopenia under TP therapy ranges from 3% in Caucasians (18) to 40% in Koreans (45), probably due to polymorphisms in genes responsible for TP metabolism (46). TPMT polymorphisms are less common in Asians than in Caucasians (47); even when leukopenia rates are higher, therefore genotyping TPMT in Asians is not so useful (48). Moreover, even in Caucasians, only a small part of myelotoxicity can be explain by TPMT polymorphisms (49), implying that other genes could play an important role in the development of toxicity. Recent studies suggest that genes like NUDT15 and FTO are associated with some cases of myelotoxicity, especially in Asians, although this association has also been identified in Caucasians (50).

Despite of the fact that the mechanism of action of NUDT15 is not well understood, it is probably responsible for the inactivation of TGNs (49), participating in the degradation of TGTP into TGMP, avoiding the incorporation of thioguanine nucleotides into cellular DNA and reducing some effects of TP (51). NUDT15 variants associated with low enzymatic activity do not change the total amount of TGNs but modify the ratio of TGTP and TGMP (49); therefore monitoring the levels of TGTP and TG integrated into the DNA could be useful to adjust the dose of TP in patients with NUDT15 deficiency (52). Although measurement of individual 6TGNs–6TGMP, 6TGDP, and 6TGTP- would be preferable as it is mentioned, these assays are not widely available.

Patients with genetic variants of NUDT15 resulting in low activity are at risk of developing toxicity, even those with intermediate activity. In Koreans, there is a variation in NUDT15 (rs116855232, mainly called p.Arg139Cys or c415C>T) that has a 89.4% sensitivity and 93.2% specificity for TP leukopenia (53). Studies suggest that virtually all patients homozygous for p.Arg139Cys will develop severe leukopenia; however, other diplotypes could also result in low enzymatic activity. Therefore, testing for p.Arg139Cys could be useful to avoid the treatment in homozygous; however, if we want to find the ideal initial dose of TP for each patient, the study of diplotypes may be necessary (54). In this way, the p.Val18_Val19insGlyVal allele is another variant of NUDT15 associated with low enzymatic activity (49). Homozygous or compound heterozygous for high-risk variants of NUDT15 are more likely to develop toxicity than those carrying intermediate or normal activity diplotypes. There are many possible combinations of these alleles and the clinical consequences can be very different, so the application of NUDT15 activity in the TP algorithm is challenging.

In Japan almost 25% of population carry one copy of the NUDT15 risk alleles and 2% are homozygous or compound heterozygous (52). The ethnic distribution of p.Arg139Cys varies from 9.8% in East Asians to 0.2% in Europeans (55). Clinical relevance also varies between populations; for example, in Chinese the risk variants of NUDT15 are supposed to be one of the most relevant in the development of toxicity (51); however, in Guatemalans these variants are not so important (49). These results suggest that ethnicity is probably one of the most important risk factors for developing toxicity under TP treatment.

A randomized controlled trial in Koreans analyzed the influence of genotyping NUDT15 before starting treatment (56). In heterozygous, they used 50 mg of AZA and, in homozygous, the treatment with TP was avoided. The rest of patients (wild type and controls) followed a stepped dose strategy. In this study, genotyping before starting treatment decreased the risk of toxicity (HR = 0.37; 95% CI: 0.18–0.77; *p* = 0.008). These results encouraged the authors to propose a similar algorithm to manage TP therapy more efficiently based on NUDT15 genotyping. They strongly recommend avoiding TP in NUDT15 homozygous.

##### Other Genetic Polymorphisms

In Asians, a polymorphism of FTO gene (rs79206939 or Ala134Thr) has been associated with a 65% decrease in its enzymatic activity and an increased risk of leukopenia (56, 57), while another polymorphism of this gene (rs16952570 CC genotype) could have a protective impact on the risk of myelotoxicity (58). Moreover, the class II HLA rs2647087 polymorphism has been associated with an increased risk of pancreatitis (59, 60). In homozygous (C/C) the risk is significantly higher (14.63%), than in heterozygous (A/C) (4.25%) or wild type (A/A) (0.53%); therefore, some authors recommend avoiding TP in homozygous and, if possible, also in heterozygous (60).

As previously mentioned, the inhibition of Rac1 activation leads to T-cell apoptosis. The Rac 1 rs34932801 polymorphism has been associated with a poor response to thiopurine therapy (61). There are also inconclusive data regarding inosine triphosphate pyrophosphatase (ITPA) polymorphisms [94C > A (rs1127354) and IVS2 + 21A>C] resulting in low enzymatic activity leading to increased 6-TGN levels and thiopurine-related toxicity (31, 62, 63).

#### Other Personal Risks

##### Age

Since, in some studies, up to 20% of patients are diagnosed of IBD at age 60 or older, new treatments improve survival and the aging of population, an increase in the number of elderly patients with IBD is expected (64). In this subgroup of patients there is concern about starting treatment with immunomodulators and biologics due to the theoretical increased risk of neoplasms, adverse events and drug interactions (65). In fact, Parian et al. found that patients older than 65 years could take an average of nine chronic drugs and, in 40% of them, there was a potential interaction between IBD therapy and chronic treatment (66).

In a recent study, patients who started TP over 60 years of age had significantly more adverse events (43.4 vs. 29.7%; *p* < 0.01), infections (3.6 vs. 2.0%; *p* < 0.001) neoplasms (1.5 vs. 0.2%; *p* < 0.001), myelotoxicity (14 vs. 7.6%, *p* < 0.01), hepatotoxicity (9 vs. 4.7%, *p* < 0.001) and digestive intolerance (12.3 vs. 10%; *p* = 0.002), than the younger ones (65). In another study, the risk of malignancy and mortality was higher in elderly patients treated with TP than in those with anti-TNF therapy (H= 3.017; 95% CI: 1.050–8.666; *p* = 0.0403 and HR= 3.682; 95% CI: 1.192–11.377; *p* = 0.0235, for malignancy and mortality, respectively) (67). Regarding neoplasms, in the prospective observational CESAME study, the rate of urinary tract cancer in patients receiving TP was 0.48/1,000 patient-years (68). Male sex (HR = 3.98, *p* = 0.04) and age over 65 years (HR = 13.26, *p* = 0.0001) were the main risk factors.

##### Viral Infections (Epstein-Barr Virus and Cytomegalovirus Serological Status)

The primo-infection of Epstein-Barr virus (EBV) in young patients treated with TP has been associated with an increased risk of HLH (69). There are also some case reports about the relationship between cytomegalovirus (CMV) and HLH or severe primo-infection in patients under thiopurine therapy (70, 71). In a recent study, exposure to TP was an independent risk factor for developing serious viral infections, mainly caused by CMV or EBV (72). After EBV infection, the virus can be reactivated and, under normal conditions, the T-lymphocytes can neutralize it; however, under TP therapy, these lymphocytes are unable to act and an uncontrollable proliferation could lead to the development of complications (69). A Spanish group found that 97.4% of IBD patients were EBV-IgG positive and, among the negative ones, the seroconversion rate was 29.7% during 4 years of follow-up, without differences between young and elderly patients; therefore, EBV serological status should be assessed, regardless of age or sex, in all patients before starting TP (69).

In a meta-analysis, the risk of lymphoma was higher in patients treated with combination therapy than in those with TP o anti-TNF monotherapy (RR= 1.10; 95% CI: 1.03–2.81; *p* = 0.039) (73). It could be explained by an additive effect: TP affect the activity of T-lymphocytes, anti-TNFs decrease the action of natural killers and both help to propagate the lymphoblastoid transformation initiated by EBV (73, 74). In addition, the results of a recent study suggest that the risk of EBV-related lymphoma could be increased in patients with low thiopurine therapy compliance, since under the treatment there is an inhibition of cell replication, but after discontinuing it, an increase in the number of B-cells and lymphoblastoids can led to uncontrollable proliferation (74). Patients with exposure to TP have a five times higher risk of lymphoproliferative disorders than those who are not treated with these drugs; however, the 10-years risk of developing these disorders in young patients is <1% (75). Moreover, in a recent study, the incidence of acute myeloid leukemia and/or myelodysplastic syndrome was 18.7 per 100,000 IBD patients-year (76). The risk was increased in patients with current TP therapy but not in those with previous exposure. Based on the data above, ECCO guidelines recommend assessing EBV serological status before starting immunosuppressive therapy (77) and balancing the risk-benefit and the availability of other compounds we recommend avoiding thiopurines in EBV-IgG negative patients, particularly in young males. Despite the increased risk of lymphoproliferative disorders associated with TP, it is important to note that these drugs have also been associated with a reduction in the incidence of colorectal cancer, due to different mechanisms not only related to its anti-inflammatory effect (78).

### Using Low Dose Thiopurine and Allopurinol

Allopurinol is a xanthine oxidase inhibitor and when added to TP increases its metabolism through 5-IMPDH into 6-TGN. LDTA can be useful in “hypermethylators” (79), in which TGN levels are usually <230–400 pmol/8 × 10^8^ and/or 6-MMP levels are over 5,700–6,000 pmol/8 × 10^8^ (80), so that they are at risk of hepatotoxicity and refractoriness to TP (81).

The increase in the levels of active metabolites can lead to myelotoxicity; therefore a 25–50% reduction in the dose of TP has been proposed to prevent it (82). In some studies, the dose of allopurinol ranges from 50 to 100 mg per day (80), however recent evidence suggests a clinical benefit without increasing toxicity using 100 mg (83). Some authors also suggest adjusting the dose of combination therapy based on TGN levels 4 weeks after starting treatment, 4 weeks after any dose change and every 6–12 months (81).

The efficacy of LDTA in non-responders and also in patients with TP-related hepatotoxicity has been demonstrated (81, 83, 84). In a recent randomized clinical trial, clinical response to LDTA was observed from week 2 of therapy, suggesting a faster onset of action, probably due to a rapid increase in TGN levels (83). Allopurinol is usually well-tolerated, with no major side effects, with skin rashes and gastrointestinal symptoms being the most common. Toxic epidermal necrolysis has been described in Asians (81).

### Adjusting Dose of TP Depending on Metabolites

The measurement of TP metabolites (TGN and MMP) can be useful to assess compliance, identify patients at risk of adverse events and also to manage refractory patients as shown in Table 2 (31); however, the efficacy and toxicity thresholds are still unclear (42).

**Table 2:** Thiopurine monitoring based on metabolites.

**6-TGN**	**6-MMP**	**Cause**	**Consequences**	**Recommendation**
Normal or high	Low	Therapeutic dose Refractoriness if absence of response	Control of disease activity No control of disease activity	Continue therapy Change therapy
High	High	Overdose	Myelotoxicity Hepatotoxicity	Reduce dose
		Refractoriness if absence of response	No control of disease activity	Change therapy
High	Low	Low TPMT activity	Risk of myelotoxicity	Reduce dose
		Refractoriness if absence of response	No control of disease activity	Change therapy
		Response	Control of disease activity	Continue monitoring
Low	High	Hypermethylators	No control of disease activity Hepatotoxicity	Reduce dose (25–50%) and add allopurinol
Low	Low	Underdose No compliance	No control of disease activity	Increase dose Assess adherence

*6-TGN levels: low (<230–235 pmol/8 × 10^8^), high (> 450 pmol/8 × 10^8^), normal (230–450 pmol/8 × 10^8^)*.

*6-MMP levels: high (>5,700 pmol/8 × 10^8^), low (<5,700 pmol/8 × 10^8^)*.

It has been suggested that the TGN target levels are likely to depend on the situation. When TP are used as monotherapy, TGN levels above 230–235 pmol/8 × 10^8^ RBC have been associated with clinical response, and more than 450 pmol/8 × 10^8^ RBC have been associated with an increased risk of myelotoxicity (5, 85). Regarding mucosal healing, a cutoff level of 397 pmol/8 × 10^8^ RBC has been proposed with high specificity but low sensibility (86.7 and 35.3%, respectively) (86). In Chinese patients, a cutoff point between 180 and 355 pmol/8 × 10^8^ RBC has been associated with remission (87). If the goal is to decrease immunogenicity related to anti-IFX antibodies, TGN levels ≥ 120 pmol/8 × 10^8^ RBC appear to be enough to significantly reduce antibodies (88). In other studies, a similar cutoff (105–125 pmol/8 × 10^8^ RBC) was also associated with maintenance of therapeutic IFX levels (89, 90).

High levels of MMP have been associated with thiopurine-related hepatotoxicity (91–93). Moreover, in one study, patients with MMP levels between 3,615 and 5,700 pmol/8 × 10^8^ RBC had a 4-fold risk of hepatotoxicity (85); however, subsequent studies did not confirm this association (21). In fact, in one study almost 90% of patients with high concentration of MMP did not develop hepatotoxicity and, in 40% of patients with hepatotoxicity, MMP levels were below the risk cutoff (21). Therefore, it seems that high levels of MMP should be associated with other alterations, such as hypertransaminasemia, to be considered a marker of hepatotoxicity (94).

The overall benefit of routine metabolite monitoring remains unclear (42), since some studies do not find a benefit (95, 96) while others consider it as a strategy to optimize TP before switching to biologics (91, 97, 98). Data on the cost-effectiveness of this strategy is also insufficient (42).

### Using Thioguanine

TG is a thiopurine with a simpler metabolism than AZA or MP. In a single pathway by HPRT, TG is transformed into TGNs, thus the methylated products associated with toxicity are not produced (99). Moreover, an experimental study in mice suggests that the effects of TG do not depend only on lymphocyte inactivation, because TG can be transformed into TGNs by the local action of colonic mucosal cells and colonic microbiota, leading to autophagy and intracellular destruction of bacteria (100). In fact, TGNs appear to accumulate in areas of intestinal inflammation, which explains the faster onset of the effect and could decrease lymphocyte-related myelotoxicity.

In terms of efficacy, ~65% of patients previously treated with AZA or MP, have clinical response with TG (101) and, in one study, in 57% of patients the addition of TG to anti-TNF therapy led to an improvement in the clinical situation (102). Efficacy seems to be similar to LDTA and discontinuation rates due to adverse events do not differ from conventional TP (16, 103). However, the main limitation of TG has been the risk of hepatotoxicity, especially the risk of nodular regenerative hyperplasia, which may be dose-related, since high doses of TG (40 mg/day) have been associated with liver injury (104), whereas studies using lower doses (20 mg/day) did not find a significant risk of this side effect and the efficacy was similar (99, 105–108). Furthermore, in another study the development of biopsy-diagnosed nodular regenerative hyperplasia was not associated with significant clinical consequences in most patients (109).

## Indications of Thiopurines in IBD in the Biologic Era

### Thiopurines as Monotherapy

#### Evidence in Crohn's Disease

TP as monotherapy seem to be inefficient for induction of clinical remission in active luminal CD and more recent clinical guidelines suggest against their use for this indication based on very low-quality evidence. Five placebo-controlled studies involving 380 patients have evaluated TP in this indication using validated outcomes measures (CDAI <150 or HBI ≤ 3). The use of concomitant steroids was allowed in four of them. The pooled analysis (intention-to-treat basis) showed no differences between TP and placebo for induction of remission (48 vs. 37%, RR= 1.23; 95% CI: 0.97–1.55) (110–114). Three trials have evaluated clinical response (not using validate outcomes measures) and no differences were found between TP and placebo (RR= 1.87, 95% CI: 0.44–7.96). Heterogeneity was serious in this case (*I*^2^ = 69%) and imprecision very serious due to sparse data and wide confidence intervals; being the quality of evidence very low for this outcome (115–117).

However, effectiveness of TP as maintenance treatment for steroid-dependent luminal CD has been consistently proven. A 2015 Cochrane systematic review, which included six trials published between 1971 and 2013, showed that AZA was superior to placebo for the maintenance of remission (73% of patients treated with AZA remained in remission compared to 62% of those who were treated with placebo, RR= 1.19; 95% CI: 1.05–1.34). Probably these figures underestimate the efficacy because the meta-analysis included studies with infra-therapeutic doses (<2 mg/kg/day), but also is important to note that the number of patients included was modest (489) and follow up was limited (6–18 months) (11). The effect of TP on fistula healing in complex perianal CD has been reported in RCT in very few patients and, therefore, there is no evidence that support its use as monotherapy in this scenario. Very interesting is a recent large-scale study whose results suggest a re-evaluation of the place for TP monotherapy in the maintenance treatment algorithm in CD. *Stournaras et al*. study assessed the long-term effectiveness of TP monotherapy with the intention of maintaining medically induced remission in 11,928 patients (4,968 UC, 6,960 CD). TP were effective, without the need for escalation to biologic therapy or need for surgery, in both UC and CD, but its efficacy was significantly lower in CD patients than in UC patients (34.2 vs. 52.7%) (118). The data summarized by Verstockt et al. on the Leuven hospital experience are less hopeful. Among 780 patients included with CD, only a small proportion of patients (7.5%, 59 patients) continued TP monotherapy till final follow-up (median of 13 years), suggesting that even in this widely accepted indication its long-term role is limited (119).

Another common use of TP is prevention of post-surgical relapse in CD. There is moderate certainty evidence that AZA and MP are superior to placebo. According to a very recent systematic review and meta-analysis (2019), after a follow-up of 12–36 months, 51% of patients treated with AZA/MP relapsed compared to 64% of patients with placebo (RR= 0.79; 95% CI 0.67–0.92; 408 participants; three studies; IR = 0%). Compared to anti-TNF drugs, TP seem to be inferior in this scenario but quality of evidence is very low. Cochrane review shows that after a follow-up of 12–24 months, 43% of participants treated with AZA clinically relapsed vs. 14% of patients in the anti-TNF group (RR= 2.89, 95% CI 1.50–5.57, 139 participants, three studies, IR = 0%) (120).

Finally, a question that has been raised in the last decade is if the early introduction of TP could modify disease course. Two studies have evaluated this point: the AZTEC and the RAPID trials (8, 121). RAPID trial compared early AZA use to classical step-up therapy in patients with risk factors for serious CD, and the AZTEC trial compared AZA with placebo up to week 76 at inducing sustained steroid-free remission in recently diagnosed uncomplicated CD. Both studies showed no effect of early AZA, which seems to argue against its early use. However, there are some caveats, including discrepancies in disease severity between groups and outcome definitions. Interestingly, in the RAPID trial, early AZA was associated with a significant reduction in new perianal fistula and a *post hoc* analysis of the AZTEC showed significantly lower rate of moderate to severe CD relapse with early AZA therapy (12 vs. 30%). Hence, the data are not completely clear on the effects of the timing of TP initiation, but delaying initiation until irreversible complications is unlikely to maximize their benefit.

#### Evidence in Ulcerative Colitis

In a meta-analysis that compared TP with placebo and/or salicylates in induction of remission in UC flare, differences were not found (122). Conversely, some observational studies have reported remission rates up to 65% (CI 95%: 55–75%) which suggest a possible efficacy in this indication. There is solid evidence about the fact that TP requires a minimum of time to obtain efficacy (at least 1 month, in most cases more than two). Because of that, such a long latency is not acceptable when patients have a flare; current guidelines do not recommend the use of TP monotherapy as inductors of remission in UC flare (123).

Maintenance of clinical remission after a mild/moderate flare in patients with steroid-dependent/steroid-resistant UC is one of the main indications of TP. Its efficacy in this scenario has been evaluated by two meta-analysis. Gisbert et al. meta-analysis reported 60% of efficacy in controlled trials with a NNT of 5 (6 RCT included) and a 76% of efficacy in uncontrolled studies (overall OR = 2.56, 95% CI: 1.51–5.3) (122). Cochrane Institute meta-analysis that included 4 RCT with 232 patients concluded that patients treated with AZA have a lower rate of failure compared to placebo (44 vs. 65%, respectively, RR = 0.68; IC 95%: 0.54–0.86) (12). It is necessary to mention the only high quality randomized controlled trial available that compared AZA and mesalazine in steroid-dependent patients, showing that AZA is significantly more effective (53 vs. 21%; OR = 4.78; 95% CI: 1.57–14.5) to induce clinical and endoscopic remission and to avoid steroid requirements in the first 6 months after the flare than mesalazine (124). Additionally, probably the efficacy of TP in this trial is underestimated because it lasted only 6 months, and efficacy would not be seen in some slow TP responders. In summary, numerous observational good quality studies that include many patients followed during very long periods confirm that TP are globally effective in UC, even more than in CD (118). However, the adverse effects of TP and the efficacy and safety of mesalazine make it the choice in many patients. In steroid-dependent patients, the superiority of TP is obvious. Also, we want to mention the first RCT that compared the efficacy of infliximab monotherapy, AZA monotherapy and combination of both drugs for UC (SUCCESS trial) (15). *Panaccione et al*. showed that in anti-TNF naïve patients with moderate-severe UC, the rates of steroid-free remission were significantly higher in patients with combination therapy than either agent alone (combo 39.7% vs. infliximab 22% vs AZA 23%). Mucosal healing at week 16 was also significantly higher in combo group (62.8%) and infliximab alone group (54.6%) than in patients receiving AZA alone (36.8%).

Regarding the use of TP as maintenance treatment after severe flare, if patients were on TP treatment when severe flare occurs, subsequent maintenance with TP monotherapy after remission is very ineffective. In naïve TP patients, TP may reduce the rate of colectomy in the mid-term after severe UC flare controlled with intravenous cyclosporine, but the rate of colectomy remains very high (at least 33% a year) (125). Because of that, a more aggressive strategy by using anti-TNF drugs is more adequate to reduce the rate of colectomies to a maximum.

#### Evidence About TP Monotherapy Withdrawal

Whether TP can be safely interrupted in patients after achieving deep remission is a challenging question in daily practice. Patients and physicians have concerns about the long-term safety of these drugs. Seven RCT, three with placebo controlled, assessed the rate of relapse after immunomodulator withdrawal compared to continued therapy (126), but the total number of patients included in these trials was low (334 patients with CD and 67 with UC) and the follow-up period not very long (ranged from 10 months to 2 years). In the single study of UC patients, there were not significant differences between both strategies. However, a recent meta-analysis of these trials shows a significantly higher relapse rate after stopping immunomodulators compared to ongoing therapy (RR= 1.85, 95% CI: 1.44–2.38, *P* < 0.001, without between-study heterogeneity). In addition, at least three observational studies have analyzed this item. Relapse rates were also higher after withdrawal TP monotherapy (126). It should be noted that many of the studies on TP withdrawal are prior to biological era when alternatives were almost non-existent. In short, although evidence suggests that the relapse rates after TP monotherapy withdrawal is higher, the question that arises is whether it compensates with the long-term toxicity that they can cause in some patients. The most feared adverse event of TP is the occurrence of a lymphoma. The absolute risk is extremely low, but the risk at 2.5-fold and its result is devastating (73). Moreover, there are other risks associated with TP use undoubtedly more common as non-melanoma skin cancers. Interestingly, the risk seems to be proportional to duration of use and decreases on cessation of TP (127). Therefore, a balance of risk-benefit must be carried out individually with each patient, especially since there are other alternatives that are safer in the long-term and more effective, although more expensive. It is important not to forget that recent reports suggest that the risk of lymphoma is comparable for TP and anti-TNF drugs. Embarking on TP treatment is a long journey and clinicians should discuss with patients and decided on a case-by-case basis. If there is one undoubted thing, it is that regular monitoring should be provided to both, in patients continuing TP in the long term and in patients after TP withdrawal.

#### Authors Comments

Sometimes it is difficult to interpret the available evidence and to apply it to a specific patient. IBD specialists have been using TP for over 50 years and scientific evidence have demonstrated TP efficacy and effectiveness in the maintenance treatment of both CD and UC. In fact, population-based long-term observational studies (118) suggest that many patients may benefit from these drugs. However, TP are applicable only in a proportion of patients, because around a 25% of them have a limiting toxicity that prevents their use. Furthermore, they are only effective in a variable proportion of those who tolerate them. In fact, after a while only a small proportion of the patients, in whom they have been used, continue to be treated with TP. In addition, the risk of toxicity is real and potentially serious, including the possibility of hematological and cutaneous neoplasms. This risk does not disappear over time, and it can affect especially patients over 60 years of age, which in the immediate future will be a very important proportion in IBD patients (128). On the other hand, we have more and more alternatives, that although they are more expensive, present fewer risks than TP. Therefore, although there is still a group of patients in which TP monotherapy is a good option, it seems that TP role is going to become more and more limited, especially if the price of the alternatives decreases.

### Combination Therapy (TP Plus Biologics)

#### Evidence

*Post hoc* analysis of initial registration trials did not show differences in outcomes stratified by baseline TP treatment. However, in 2010 was published the SONIC trial which included 508 patients naïve to both anti-TNF drugs and TP with moderate to severe CD. Results of this trial showed the superiority of combination treatment (TP plus infliximab) compared to infliximab or AZA monotherapy in achieving steroid free clinical remission (56.8% vs. 44.4 and 30%, respectively) and mucosal healing (43.9% vs. 30 and 16.5%, respectively) at week 26 (9). The rates of adverse events were similar in the three arms and rather, there were significantly lower rates of serious adverse events in those patients that received combination therapy (RR= 0.56; 95% CI: 0.32–0.97). Later, the subsequent UC SUCCESS trial employed a similar design than SONIC trial but in naïve UC patients. Results of UC SUCCESS trial were also in favor of combination therapy. Combo-therapy was more effective than either agent alone in inducing clinical remission at week 16 and more effective than AZA monotherapy in reaching mucosal healing (62.8 vs. 36.8%, *p* = 0.001). However, there was no significant difference in the rates of mucosal healing observed with combination therapy vs. infliximab monotherapy (62.8 vs. 54.6%; *p* = 0.295) (15). In both trials, AZA co-therapy dramatically reduced the formation of anti-infliximab antibodies (in SONIC 0.9 vs. 14.6% and in SUCCESS 3 vs. 19%). SONIC trial also showed an increase in IFX median trough concentrations at week 30 in the combination arm (3.5 vs. 1.6 micgr/ml, *p* < 0.001). Conversely, for adalimumab and azathioprine, the DIAMOND trial (only 176 patients with CD) showed rates of clinical remission similar between monotherapy and combination therapy and although the rate of mucosa healing at 26 weeks was superior in combo group, this benefit was not sustained at 1 year (129). Of note, the dose of AZA used in this trial was lower than the usual dose used in CD patients (25–100 mg/day instead of 2–2.5 mg/kg/day).

Based on pharmacokinetic data of these trials the hypothesis emerged that infliximab is more immunogenic than adalimumab and the addition of immunosuppressive therapy confers more benefit what was reflected as higher drugs level. This hypothesis was supported by the prospective observational UK PANTS study that showed formation of anti-drug antibody was more frequent with infliximab than with adalimumab and was decreased by combination therapy (immunomodulator drug and anti-TNF) (130). Curiously, although the absolute risk of anti-drug antibody was lower with adalimumab, the relative risk reduction with the concomitant use of immunosuppression was similar for both anti-TNF drugs. Combination therapy has also been shown to raise adalimumab levels, which itself is associated with higher rates of clinical and endoscopic remission. Notably, last year (2020) was published the work of *Targownik and colleagues* that included 11,244 Canadian patients and used data from four population level health care databases (131). Authors showed that use of a concomitant immunomodulator (TP or methotrexate) at the time of anti-TNF initiation (infliximab or adalimumab) was associated with significantly reduction in the likelihood of treatment failure. The choice of immunomodulator did not show a significant effect in CD, but better outcomes were seen with AZA than methotrexate in UC. This study supports that benefits of combination therapy seen in RCT seem to extend to the real-world setting. It also supports the idea that adding an immunomodulator in adalimumab initiation can improve clinical outcome in the medium and long term.

Another commonly encountered scenario in clinical practice is patients who have failed or have had an inadequate response to TP and in whom anti-TNF therapy is started. No RCT has directly compared whether in such cases TP maintenance in combination with the anti-TNF would carry additional benefits in terms of efficacy. A *post-hoc* analysis of RCTs has shown no added benefit (132). However, immunogenicity should be considered and, in the absence of direct evidence, an individualized approach should be considered.

A new role of TP may be in case of switching anti-TNF. The addition of a TP is an effective method of managing secondary loss of response. A large retrospective study (2017) showed that the addition of an immunomodulator resulted in disappearance of antidrug antibody in 77% of patients with a subsequent increased of drug concentration and recapture of clinical response (133). Current reactive therapeutic drug monitoring-based algorithms propose that patients with secondary loss of response to anti-TNF with high titer of antidrug antibody should switch to another anti-TNF agent (134). Current evidence shows that these patients have more risk of developing antibody and secondary loss of response to a subsequent anti-TNF (135, 136). This effect seems to be able to be mitigated by the addition of a TP as demonstrated Robblin et al. in their RCT (included 90 patients with immune-mediated loss of response to a first anti-TNF in monotherapy) (137).

#### Authors Comments

Evidence shows that combination therapy (TP plus anti-TNF) is superior to monotherapy in treating naïve CD and UC patients, mainly due to the effect of TP on immunogenicity but also partly due to an additive immunosuppressive effect. However, before starting combination therapy, a patient-stratified risk of combination therapy-related serious adverse events (special attention to the risk of lymphomas and cutaneous neoplasms with long-term therapies and infections) must be done and a regular monitoring should be provided to these patients. Emerging data suggest that an “optimize anti-TNF monotherapy” using proactive monitoring drug levels to ensure adequate circulating anti-TNF concentrations is associated with higher rates of clinical and endoscopic remission (138, 139). This strategy may obviate in the future the need for combination therapy, but a pragmatic trial comparing both strategies has not yet taken place. In any way, in patients in whom monotherapy is chosen, we think that a proactive drug monitoring strategy is advisable. Moreover, testing patients for HLA-DQA1 ^*^ 05, which according to recent evidence is associated with an increased risk of development of antibodies against anti-TNF drug, might help physicians decide if patients should be treated with anti-TNF alone or combination therapy (140).

### Maintenance After Combination Therapy

When a patient starts combined treatment there is always a concern about how long the patients should take both treatments. Current clinical guidelines suggest maintenance with the same biologic agent in monotherapy after achieving remission with combination therapy (anti-TNF plus TP) (3, 123). There are conflicting data as to whether continuing TP beyond a certain time provides additional clinical benefit. One study has shown that, in most cases, immunization occurs during the first 12 months of anti-TNF, suggesting that to lengthen the combo-therapy further in many patients may be no necessary (141). A recent meta-analysis including a total of 186 patients with IBD in remission on combination therapy with TP plus anti-TNF (infliximab or adalimumab) analyzed the relapse rate after TP stopping (126). No statistically significant difference was observed between the groups (RR = 1.30, 95% CI: 0.81–2.08, *p* = 0.269; *I*^2^ = 0.0%, *p* = 0.641). Van Assche et al. suggested that AZA can be withdrawn after 6 months of remission on combination infliximab/AZA but many patients were on AZA for > 6 months prior to AZA withdrawal and continuation of combination therapy was associated with lower levels of serum C reactive protein and higher infliximab trough levels (142). In the study of Roblin et al., three strategies were compared: continuing with both treatments, stopping TP and decreasing TP dose (90). There were no clinical significantly differences between the strategies, but authors concluded that reducing the dose of TP was associated globally (considering levels of infliximab, antidrug antibodies and unfavorable evolution) with a better outcome than treatment stopping. In combination therapy, a reduced dose of TP may reduce the production of neutralizing anti- TNF antibodies, thereby providing a lower chance of developing adverse events. Although TP withdrawal from combination regimen carries a higher risk of anti-drug antibody formation, their effect on clinical outcomes may take longer than a year to become apparent. Meta-analysis of nine studies on adalimumab by Chalhoub et al. (after data included were re-analyzed) did not reveal any differences in maintenance clinical remission (RR= 1.01, 95% CI: 0.91–1.13) between combo and monotherapy (3, 143). In the ADHERE cohort, an open label extension study of patients included in CHARM study on adalimumab, the rates of clinical remission were similar in patients with and without concomitant immunomodulators at baseline after 3 year of follow up (144). In addition, several observational studies have investigated the risk of relapse in IBD patients who received combination treatment followed by discontinuation of immunomodulator. Two French retrospective observational studies have documented fewer flares, fewer perianal complications and fewer switching with combination therapy for > 2 years (145, 146). Two studies of CD and one of UC reported that a shorter duration of combo-treatment was associated with an increased risk of treatment failure after withdrawal of TP. The thresholds were 27, 6 and 9 months, respectively (145, 147, 148). Another recent observational study also shows that long combo-therapy (>12 months) was not more efficacious than short combination (149).

#### Authors Comments

TP withdrawal from combination with biologics after clinical remission remains a preferred approach of long-term treatment to avoid toxicity, but balancing between adverse drug effects and disease progression is unavoidable in patients with severe inflammation and complications. In severe IBD, advantages of combination therapy can outweigh the risk of lymphoma and severe infection, but in patients with mild/moderate IBD, the risk/benefit ratio is clearly less favorable. Predictive factors of relapse and evidence of deep remission should be included in the risk/benefit analysis prior to therapy withdrawal. The need for a RCT to facilitate decision making about the exact timing and optimal group of patients to discontinue therapy is clear. Recently, SPARE trial, that addresses this issue, has been completed and its results will be known soon.

## Conclusions

Thiopurines are still useful for maintenance of remission in steroid-dependence, prevent post-surgical relapse and improve the outcomes of biologic therapy. The key is to select patients properly based on personal characteristics and the course of the disease. Moreover, as explained above, many strategies are available to improve the efficacy of these drugs and to prevent adverse events.

## Author Contributions

FG conceived the idea of the review, coordinated, supervised, and edited the manuscript. CG-P and VL performed the literature review and wrote the manuscript. All authors contributed to the article and approved the submitted version.

## Conflict of Interest

The authors declare that the research was conducted in the absence of any commercial or financial relationships that could be construed as a potential conflict of interest.
